# Supplementary material for: Plasma membrane-derived extracellular microvesicles mediate non-canonical intercellular NOTCH signaling
Source: Nat Commun. 2017 Sep 27;8:709. doi: 10.1038/s41467-017-00767-2 (PMC5617834; doi:10.1038/s41467-017-00767-2)

Supplementary Figure 1: Peptides in NOTCH2 protein identified by mass-spectrometry are highlighted. The transmembrane region of NOTCH2 is underlined.

MPALRPALLW ALLALWLCCA APAHALQCRD GYEPCVNEGM CVTYHNGTGY CKCPEGFLGE YCQHRDPCEK NRCQNGGTCV AQAMLGKATC RCASGFTGED

CQYSTSHPCF VSRPCLNGGT CHMLSRDTYE CTCQVGFTGK ECQWTDACLS HPCANGSTCT TVANQFSCKC LTGFTGQKCE TDVNECDIPG HCQHGGTCLN

LPGSYQCQCP QGFTGQYCDS LYVPCAPSPC VNGGTCRQTG DFTFECNCLP GFEGSTCERN IDDCPNHRCQ NGGVCVDGVN TYNCRCPPQW TGQFCTEDVD

ECLLQPNACQ NGGTCANRNG GYGCVCVNGW SGDDCSENID DCAFASCTPG STCIDRVASF SCMCPEGKAG LLCHLDDACI SNPCHKGALC DTNPLNGQYI

CTCPQGYKGA DCTEDVDECA MANSNPCEHA GKCVNTDGAF HCECLKGYAG PRCEMDINEC HSDPCQNDAT CLDKIGGFTC LCMPGFKGVH CELEINECQS

NPCVNNGQCV DKVNRFQCLC PPGFTGPVCQ IDIDDCSSTP CLNGAKCIDH PNGYECQCAT GFTGVLCEEN IDNCDPDPCH HGQCQDGIDS YTCICNPGYM

GAICSDQIDE CYSSPCLNDG RCIDLVNGYQ CNCQPGTSGV NCEINFDDCA SNPCIHGICM DGINRYSCVC SPGFTGQRCN IDIDECASNP CRKGATCING

VNGFRCICPE GPHHPSCYSQ VNECLSNPCI HGNCTGGLSG YKCLCDAGWV GINCEVDKNE CLSNPCQNGG TCDNLVNGYR CTCKKGFKGY NCQVNIDECA

SNPCLNQGTC FDDISGYTCH CVLPYTGKNC QTVLAPCSPN PCENAAVCKE SPNFESYTCL CAPGWQGQRC TIDIDECISK PCMNHGLCHN TQGSYMCECP

PGFSGMDCEE DIDDCLANPC QNGGSCMDGV NTFSCLCLPG FTGDKCQTDM NECLSEPCKN GGTCSDYVNS YTCKCQAGFD GVHCENNINE CTESSCFNGG

TCVDGINSFS CLCPVGFTGS FCLHEINECS SHPCLNEGTC VDGLGTYRCS CPLGYTGKNC QTLVNLCSRS PCKNKGTCVQ KKAESQCLCP SGWAGAYCDV

PNVSCDIAAS RRGVLVEHLC QHSGVCINAG NTHYCQCPLG YTGSYCEEQL DECASNPCQH GATCSDFIGG YRCECVPGYQ GVNCEYEVDE CQNQPCQNGG

TCIDLVNHFK CSCPPGTRGL LCEENIDDCA RGPHCLNGGQ CMDRIGGYSC RCLPGFAGER CEGDINECLS NPCSSEGSLD CIQLTNDYLC VCRSAFTGRH

CETFVDVCPQ MPCLNGGTCA VASNMPDGFI CRCPPGFSGA RCQSSCGQVK CRKGEQCVHT ASGPRCFCPS PRDCESGCAS SPCQHGGSCH PQRQPPYYSC

QCAPPFSGSR CELYTAPPST PPATCLSQYC ADKARDGVCD EACNSHACQW DGGDCSLTME NPWANCSSPL PCWDYINNQC DELCNTVECL FDNFECQGNS

KTCKYDKYCA DHFKDNHCDQ GCNSEECGWD GLDCAADQPE NLAEGTLVIV VLMPPEQLLQ DARSFLRALG TLLHTNLRIK RDSQGELMVY PYYGEKSAAM

KKQRMTRRSL PGEQEQEVAG SKVFLEIDNR QCVQDSDHCF KNTDAAAALL ASHAIQGTLS YPLVSVVSES LTPERTQLLY LLAVAVVIIL FIILLGVIMA

KRKRKHGSLW LPEGFTLRRD ASNHKRREPV GQDAVGLKNL SVQVSEANLI GTGTSEHWVD DEGPQPKKVK AEDEALLSEE DDPIDRRPWT QQHLEAADIR

RTPSLALTPP QAEQEVDVLD VNVRGPDGCT PLMLASLRGG SSDLSDEDED AEDSSANIIT DLVYQGASLQ AQTDRTGEMA LHLAARYSRA DAAKRLLDAG

ADANAQDNMG RCPLHAAVAA DAQGVFQILI RNRVTDLDAR MNDGTTPLIL AARLAVEGMV AELINCQADV NAVDDHGKSA LHWAAAVNNV EATLLLLKNG

ANRDMQDNKE ETPLFLAARE GSYEAAKILL DHFANRDITD HMDRLPRDVA RDRMHHDIVR LLDEYNVTPS PPGTVLTSAL SPVICGPNRS FLSLKHTPMG

KKSRRPSAKS TMPTSLPNLA KEAKDAKGSR RKKSLSEKVQ LSESSVTLSP VDSLESPHTY VSDTTSSPMI TSPGILQASP NPMLATAAPP APVHAQHALS

FSNLHEMQPL AHGASTVLPS VSQLLSHHHI VSPGSGSAGS LSRLHPVPVP ADWMNRMEVN ETQYNEMFGM VLAPAEGTHP GIAPQSRPPE GKHITTPREP

LPPIVTFQLI PKGSIAQPAG APQPQSTCPP AVAGPLPTMY QIPEMARLPS VAFPTAMMPQ QDGQVAQTIL PAYHPFPASV GKYPTPPSQH SYASSNAAER

TPSHSGHLQG EHPYLTPSPE SPDQWSSSSP HSASDWSDVT TSPTPGGAGG GQRGPGTHMS EPPHNNMQVY A

Supplementary Figure 2: (a) Western blotting results showing the presence of NOTCH2 short form in extracellular vesicles from multiple cell lines (HCC1419, A549 and MCF-7 cells). (b) ARRDC1 knockdown (KD) reduces NOTCH2 in EVs in MCF-7 cells. ARRDC1 was knockdown by siRNA in MCF-7 cells. Western blotting was done to detect ARRDC1 and NOTCH2 as well as the exosomal marker CD9 in cell lysates and in extracellular vesicles (EV).

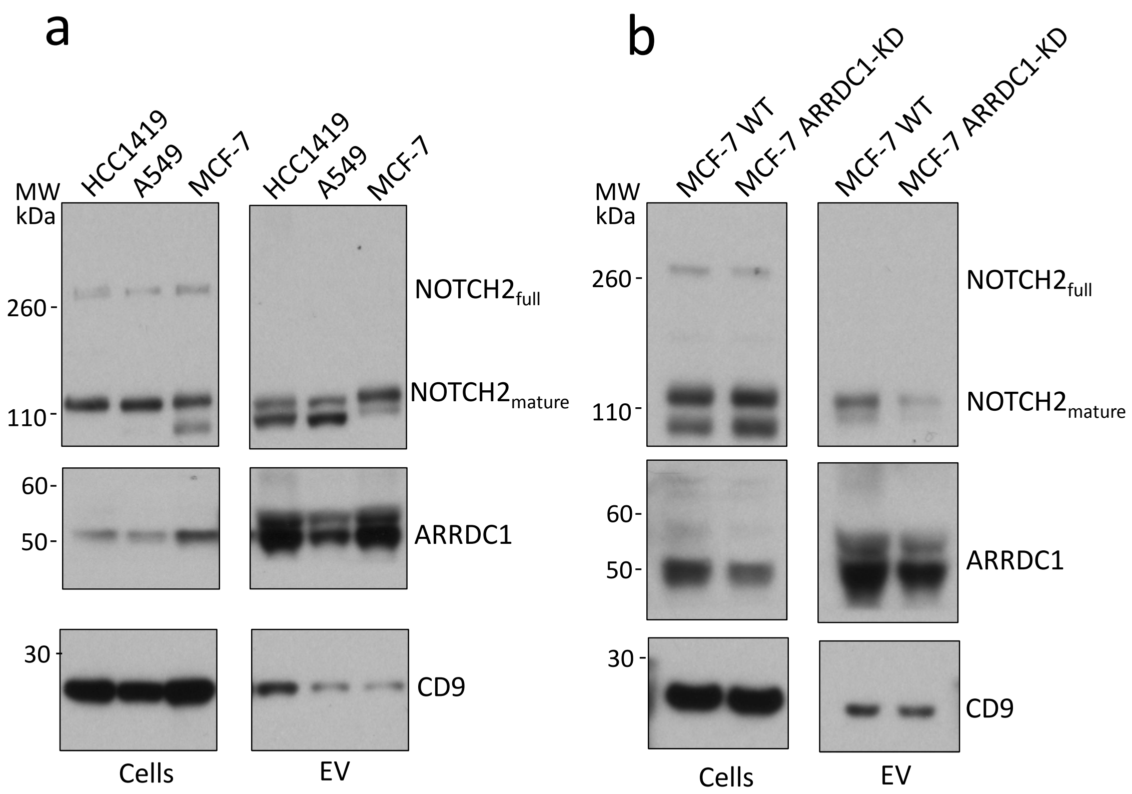

Supplementary Figure 3: Isolated ARMMs were incubated with HEK293T cells for 2 hours. After washing with PBS, cells were fixed and immunostained with indicated antibodies and fluorescence-labeled secondary antibodies. DAPI was used to stain nuclei. Confocal imaging was done using Leica SP8 X.

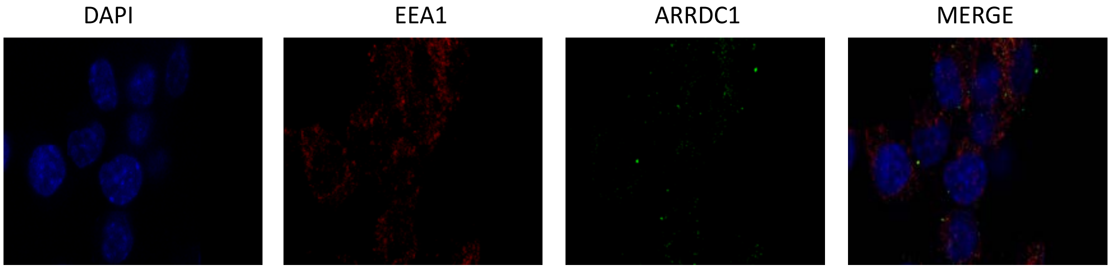

Supplementary Figure 4: Uncropped images of Western blotting gels

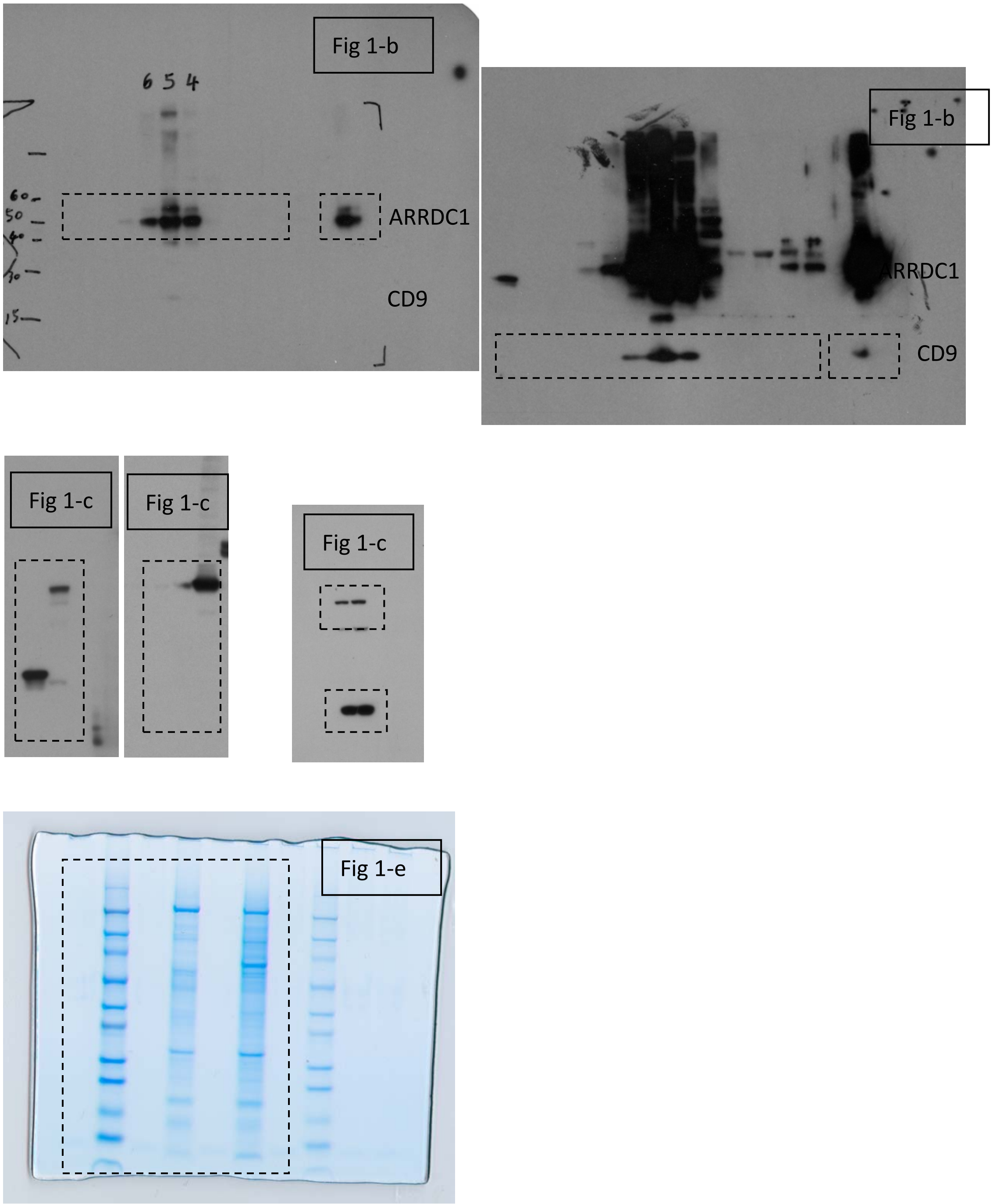

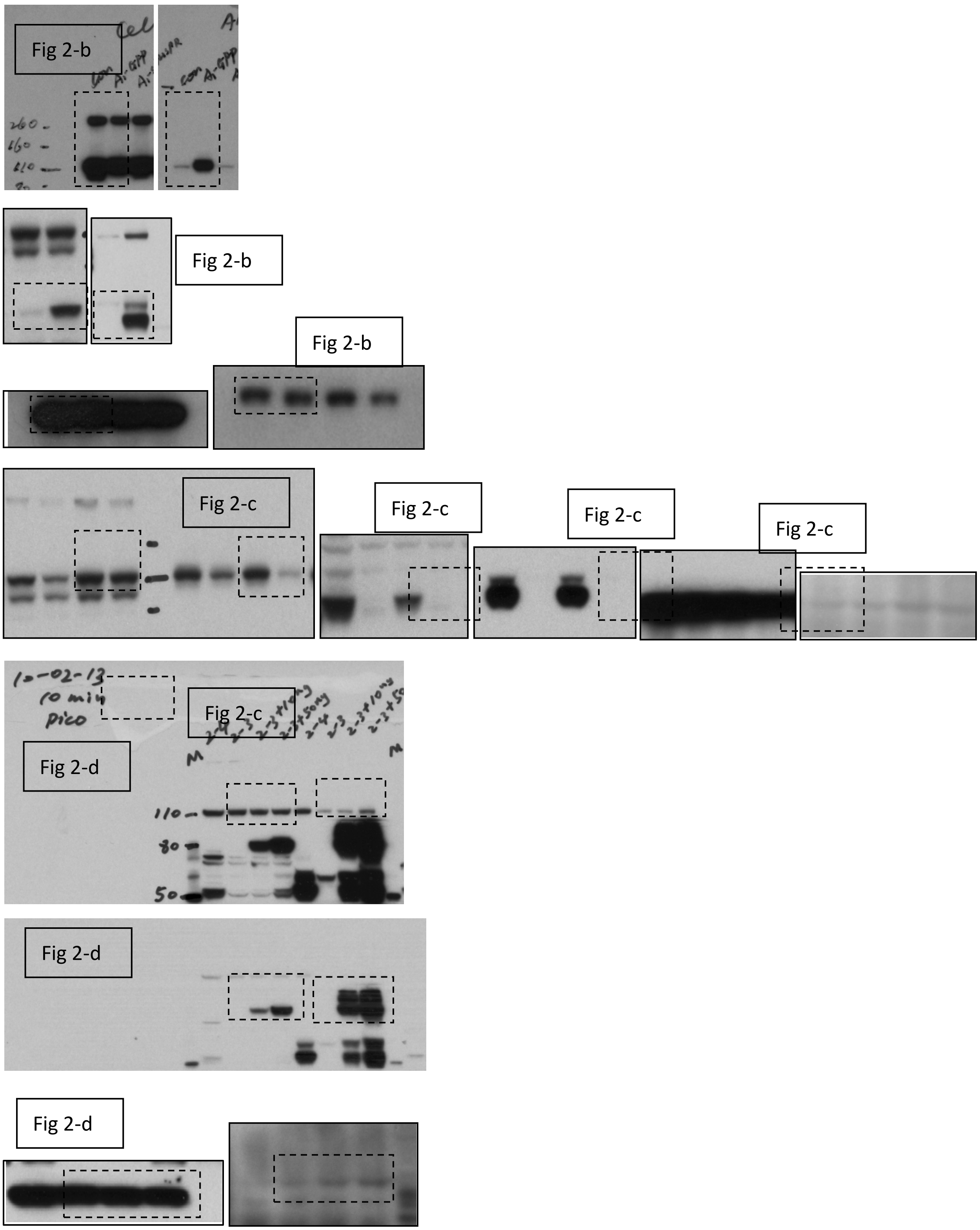

Fig 2-b
260
160
110
Fig 2-b
Fig 2-b
Fig 2-c
Fig 2-c
Fig 2-c
Fig 2-c
10-02-13
10 min
pico
Fig 2-c
Fig 2-d
M
110
80
50
Fig 2-d
Fig 2-d

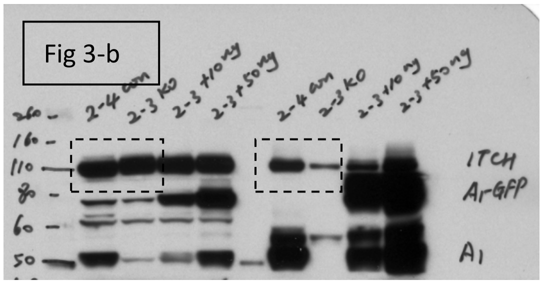
Fig 3-b
2-4 con
2-3 KO
2-3 +10 ng
2-3 +50 ng
2-4 con
2-3 KO
2-3 +10 ng
2-3 +50 ng
260
160
110
80
60
50
ITCH
A1-GFP
A1

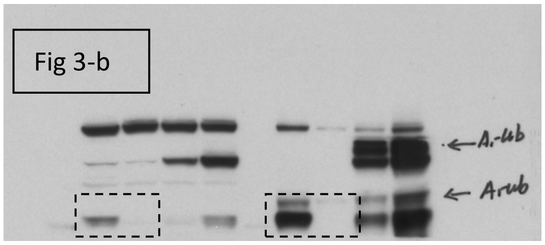
Fig 3-b
A1-ub
A1ub

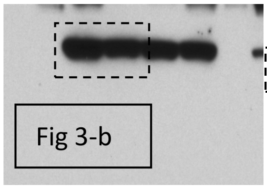
Fig 3-b

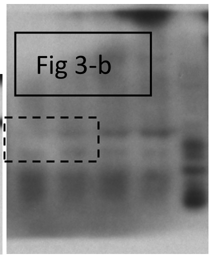
Fig 3-b

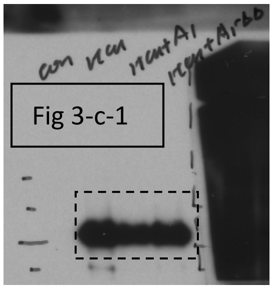
Fig 3-c-1

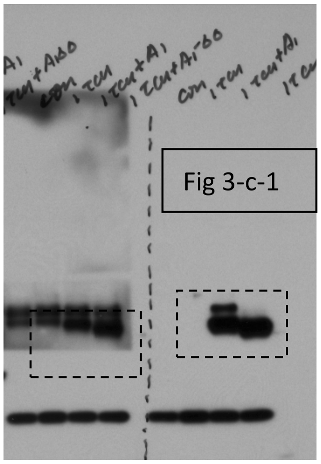
Fig 3-c-1

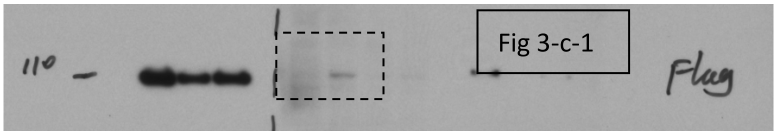
Fig 3-c-1
110
Flag

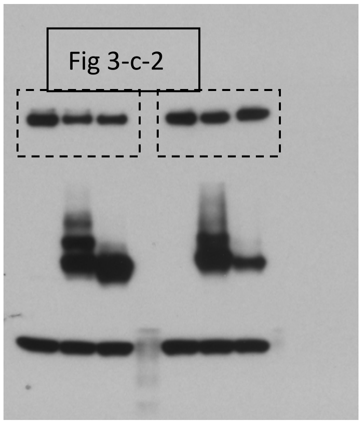
Fig 3-c-2

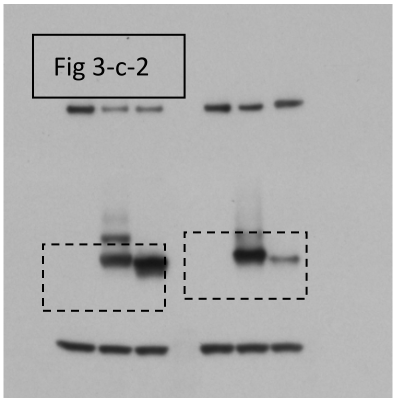
Fig 3-c-2

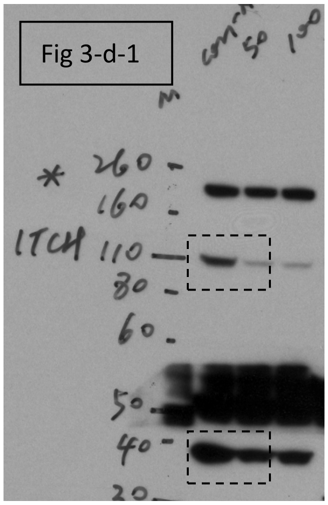
Fig 3-d-1
*
260
160
ITCH
110
80
60
50
40

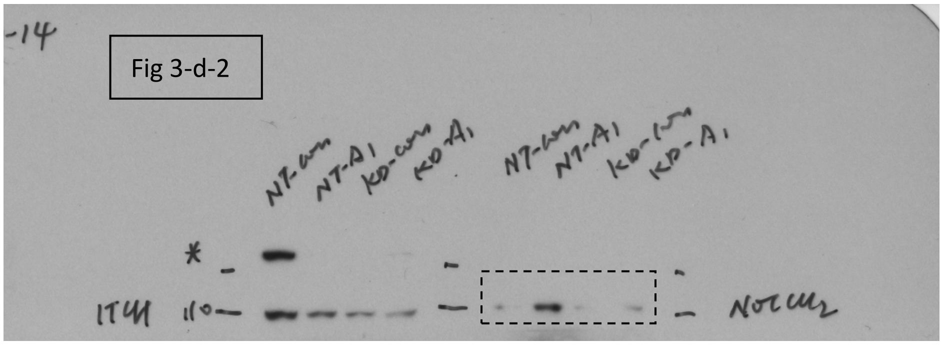
Fig 3-d-2
NT-con
NT-A1
KD-con
KD-A1
NT-con
NT-A1
KD-con
KD-A1
*
ITCH
110
Notch2

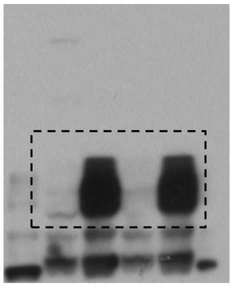

Fig 3-d-2

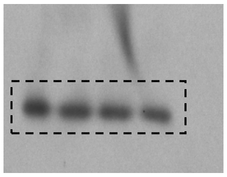

Fig 4b

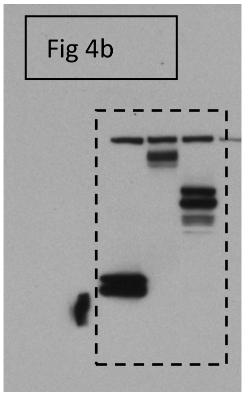

Fig 4b

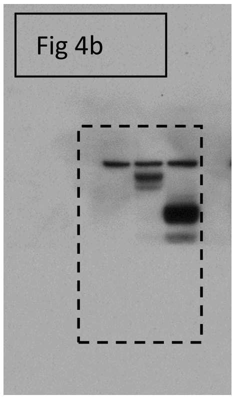

Fig 4b

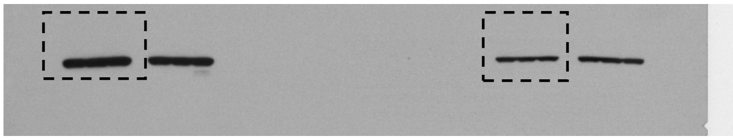

Fig 5b

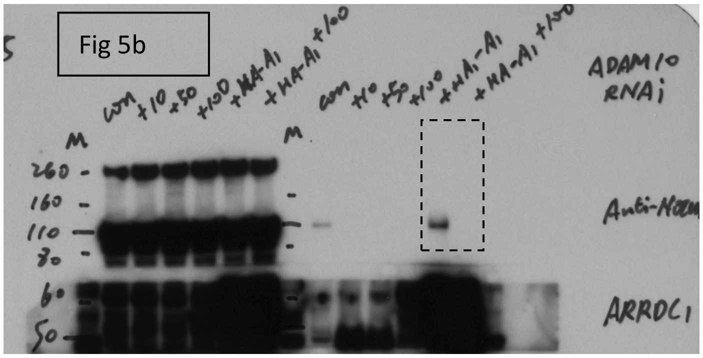

Fig 5b

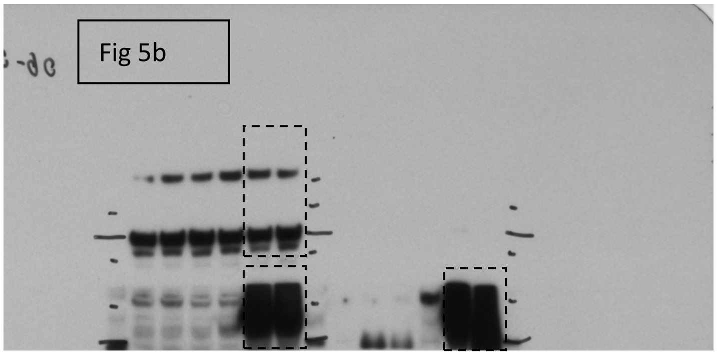

Fig S2-a

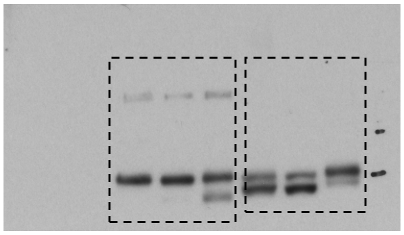

Fig S2-a

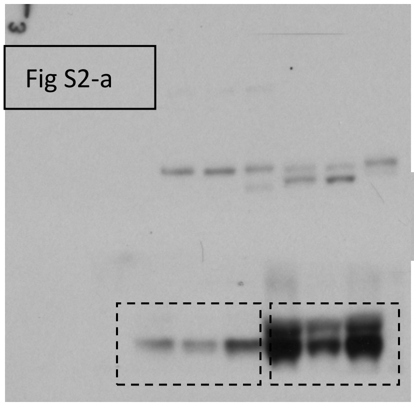

Fig S2-a

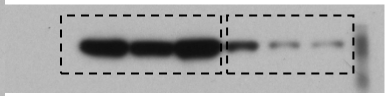

Fig S2-b

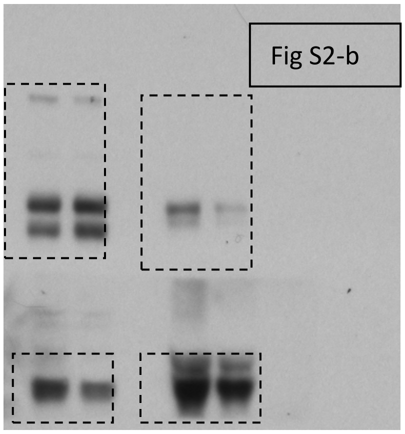

Fig S2-b

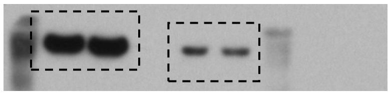

Supplement: Supplementary file 1 — Supplementary Information [file 41467_2017_767_MOESM1_ESM.pdf]
