# Supplementary material for: Plasma membrane-derived extracellular microvesicles mediate non-canonical intercellular NOTCH signaling
Source: Nat Commun. 2017 Sep 27;8:709. doi: 10.1038/s41467-017-00767-2 (PMC5617834; doi:10.1038/s41467-017-00767-2)
Supplement: Supplementary file 3 — Supplementary Descriptions [file 41467_2017_767_MOESM3_ESM.pdf]

Description of Additional Supplementary Files

File Name: Supplementary Data 1
Description: List of proteins enriched (>1.5 fold) in ARMMs.

File Name: Supplementary Data 2
Description: List of proteins with peptides detected only in ARRDC1-GFP ARMMs.

File Name: Supplementary Data 3
Description: List of membrane proteins enriched in ARMMs.

File Name: Supplementary Data 4
Description: List of ESCRT proteins enriched in ARMMs.
